# Clinical efficacy of customized modular prosthesis in the treatment of femoral shaft metastases

**DOI:** 10.3389/fonc.2023.1115898

**Published:** 2023-04-06

**Authors:** Feifei Pu, Yihan Yu, Zengwu Shao, Wei Wu, Jing Feng, Fengxia Chen, Zhicai Zhang

**Affiliations:** ^1^ Department of Orthopedics, Wuhan Hospital of Traditional Chinese and Western Medicine (Wuhan No.1 Hospital), Tongji Medical College, Huazhong University of Science and Technology, Wuhan, Hubei, China; ^2^ Departments of Orthopedics, Union Hospital, Tongji Medical College, Huazhong University of Science and Technology, Wuhan, Hubei, China; ^3^ Department of Radiation and Medical Oncology, Zhongnan Hospital, Wuhan University, Wuhan, Hubei, China

**Keywords:** femoral shaft, bone metastases, en bloc resection, customized modular prosthesis, surgical treatment

## Abstract

**Purpose:**

To examine clinical outcomes of a specialized modular prosthesis used to fill a bone deficiency following removal of femoral shaft metastases.

**Methods:**

Eighteen patients with femoral shaft metastases who underwent en bloc resection and implantation of a personalized modular prosthesis between December 2014 and December 2019 were retrospectively analyzed. Pain, limb function, and quality of life were evaluated using the visual analog scale (VAS), Musculoskeletal Tumor Society (MSTS) scale, International Society of Limb Salvage (ISOLS) scoring system, Karnofsky Performance Status (KPS) scale, and Nottingham Health Profile (NHP) scale. The Kaplan–Meier technique was used to analyze patient survival.

**Results:**

The operation duration was 90–150 min (mean, 115 min), and the osteotomy length was 9–16 cm (mean, 11.72 cm). The patients were followed for 12–62 months (mean, 25.28 months). The VAS and NHP ratings were lower at 3, 6, and 12 months after surgery than before surgery, while the MSTS, ISOLS, and KPS scores were higher after surgery than they had been before. These differences were statistically significant (*P*<0.05). The survival period was between 7 and 62 months (mean, 20.89 months), and the rates of survival at 1-year and 2-year were 72.22% and 27.78%, respectively. Except for two patients with aseptic prosthesis loosening during the follow-up period, there were no problems.

**Conclusion:**

En bloc excision and implantation of a personalized modular prosthesis can reduce pain and improve the ability of patients with femoral shaft metastases to perform daily activities, thereby improving their quality of life.

## 1 Introduction

The long bones of the limbs are frequently affected by bone metastases, and the femoral shaft is the most frequently affected site, accounting for 25% to 71% of long bone metastases, 25% of which lead to pathological fracture (1). Metastases in the femoral shaft can result in excruciating pain, limb impairment, and lower quality of life (2). Bone metastases weaken bones and cause pathological fractures, both of which are significant risk factors for death (3). The best treatment plan must be chosen to prevent and treat pathological fractures in patients with bone metastases (4).

There is a broad agreement that limb salvage surgery enhances the quality of life of patients with limb shaft metastases owing to recent advancements in radiotherapy, chemotherapy, surgical techniques, targeted therapy, and immunotherapy for comprehensive cancer treatment (5). Following the removal of a bone tumor, several reconstruction techniques can be used, each with advantages and disadvantages. These techniques include biological reconstruction, artificial articulation-allograft reconstruction, intramedullary needle fixation, plate screw fixation, and tumor prosthesis replacement (6, 7). The most popular reconstruction technique in limb salvage surgery is prosthesis replacement because it can quickly relieve pain and restore limb function, while having a low incidence of post-operative complications (8–10).

Because of their positive clinical outcomes, personalized modular prostheses have recently gained recognition as a new treatment option for femoral shaft metastases (11–13). Intercalary prosthesis implantation provides the advantages of no delayed end healing and no autogenous or allogeneic bone fractures (14–16). Early post-operative functional exercise is possible because the prosthesis has good strength and can bear significant stress, provided that the post-operative limb force line is normal. Additionally, because the prosthesis may be customized, the osteotomy plane can precisely reach the area that needs to be excised, thereby reducing the chance of local recurrence. En bloc resection and intercalary prosthesis insertion take less time during surgery when the diaphysis is being repaired following large-segment osteotomy.

However, the surgical impact, functional success, and consequences of the treatment of femoral shaft metastases are not entirely obvious owing to the short duration of clinical use. This study sought to provide 18 patients with femoral shaft metastases with an effective surgical alternative by summarizing the results of en bloc resection and installation of tailored modular prostheses.

## 2 Materials and methods

### 2.1 Ethical approval and consent to participate

This retrospective study adhered to the Declaration of Helsinki and was authorized by our school’s Ethics Committee. Our ethics committee approved the process and data collection.

### 2.2 Inclusion and exclusion criteria

The inclusion criteria were as follows: femoral shaft metastases, an expected survival time of >3 months, an effective fixation length of the remaining bone marrow cavity at both ends after osteotomy of >5 cm, pathological fractures or a Mirels score of >9, and complete data with a follow-up period of >3 months. Patients with poor general health who could not handle anesthesia or surgery were excluded.

### 2.3 Patients

In Wuhan Hospital of Traditional Chinese and Western Medicine (Wuhan No. 1 Hospital) and Union Hospital, Tongji Medical College, Huazhong University of Science and Technology, 18 patients (of which five had pathological fractures) with femoral shaft metastases were treated between December 2014 and December 2019 by employing en bloc excision and implantation of a personalized modular prosthesis. There were 11 men and seven women aged 46–79 years (median, 65.94 years) in this group. All patients’ lower limb pain and activity restrictions led them to visit the hospital. The central segment of the femoral shaft was the location of the tumor lesions in all cases. The primary tumor types were lung cancer (n = 7), kidney cancer (n = 4), breast cancer (n = 2), thyroid cancer (n = 2), cervical cancer (n = 1), colon cancer (n = 1), and stomach cancer (n = 1).

### 2.4 Prosthetic design

**Figure 1:**
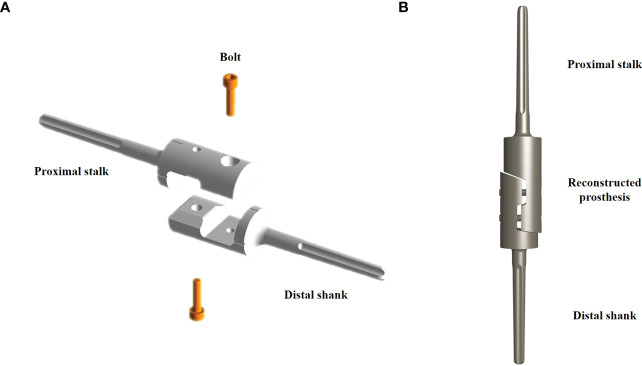
Schematic diagram of the customized modular prosthesis. **(A)** The decomposition components include the distal prosthesis stem, proximal prosthesis stem, and two intermediate screws. **(B)** Schematic diagram of the assembled intercalary prosthesis.

Magnetic resonance imaging and preoperative radiography were performed to customize the modular prosthesis, which was created and produced by Beijing Lidak Technology Co., Ltd. (Beijing, China). The distal and proximal prosthesis stems, as well as the intermediate screws, were the main parts of the prosthesis, which were made of a titanium alloy (Ti6A14V). The distal and proximal prosthesis stems were grooved, and a two-fold taper connected the implanted prosthesis to the bone (**Figures 1A, B**).

### 2.5 Surgical procedure

**Figure 2:**
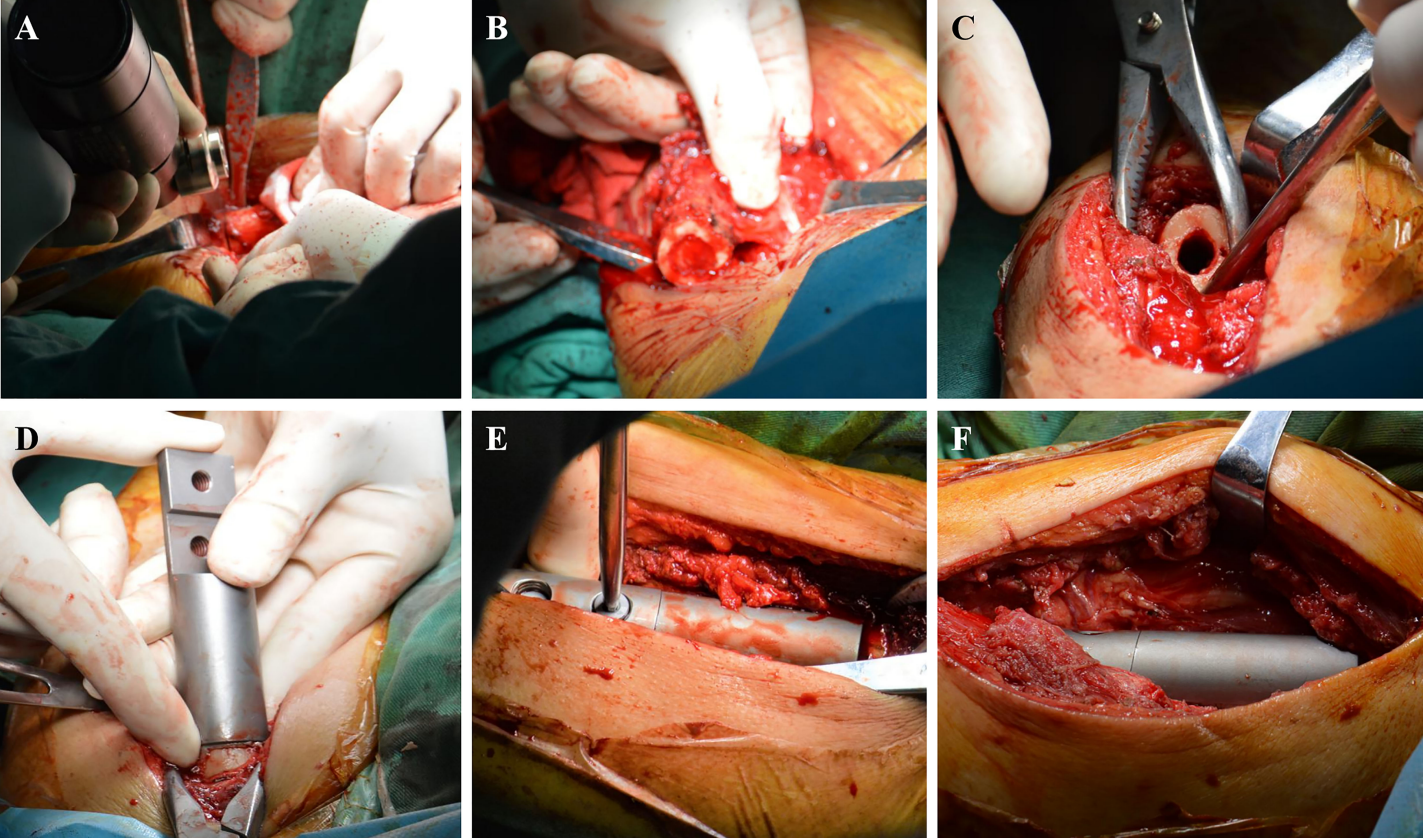
Surgical procedure. **(A)** The distal diseased bone is cut by a chainsaw. **(B)** The diseased bone is removed. **(C)** The medullary cavity is expanded. **(D)** Simulated prosthesis is installed. **(E)** The prosthesis is locked with screws. **(F)** Intercalary prosthesis is assembled.

The lateral thigh approach was used in patients who were positioned in the supine position. The length of the incision was chosen based on the degree of tumor involvement revealed on preoperative magnetic resonance imaging. Following skin and deep fascia incisions, the tumor location of the femoral shaft metastases was visible between the vastus lateralis and vastus posteris. The degree of intramedullary invasion revealed by magnetic resonance imaging was used to calculate osteotomy length and plane. The periosteum was removed at the osteotomy plane, and periosteum strippers were positioned on either side to safeguard nearby soft tissue. To complete en bloc resection, a chainsaw was used to chop the diseased bone fragment (**Figures 2A, B**).

**Figure 3:**
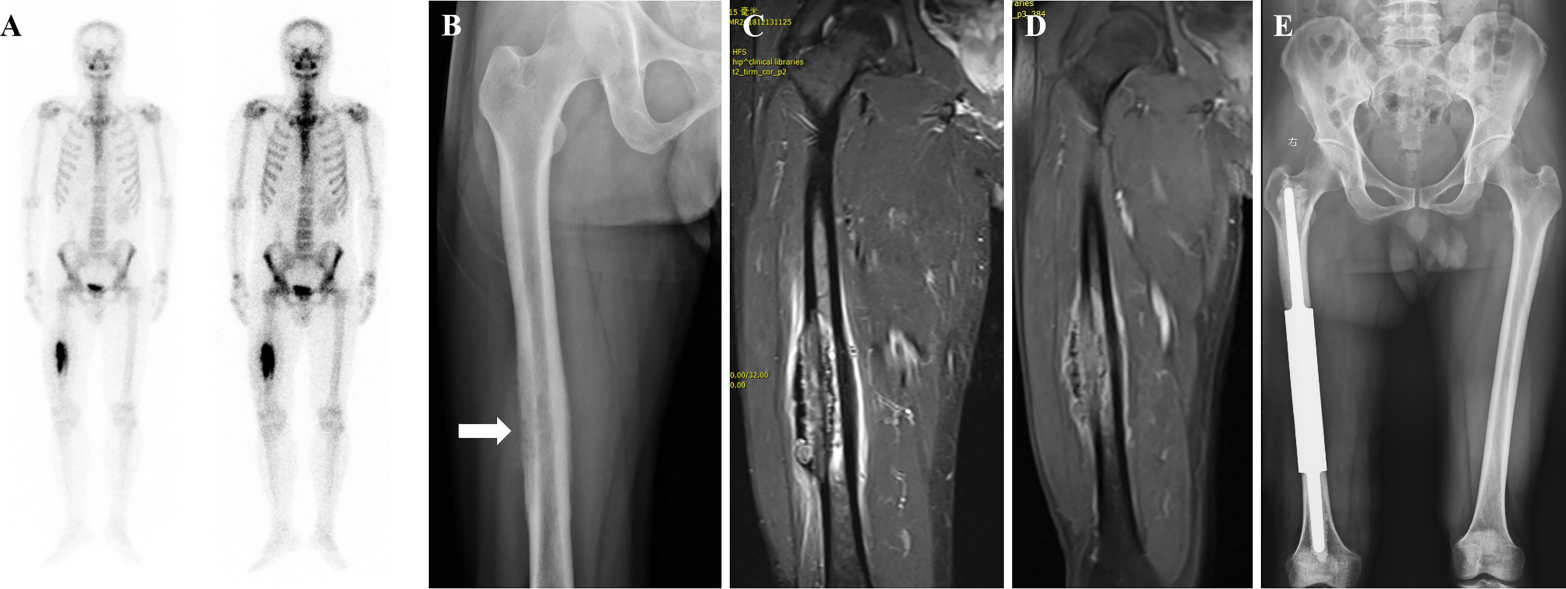
A patient with isolated metastasis of the right femoral shaft. **(A)** Emission Computed Tomography showing an isolated metastatic lesion in the right femoral shaft with active metabolism. **(B)** Radiograph showing osteolytic destruction of the right femoral shaft. **(C, D)** Magnetic resonance image showing decreased T1-weighted image signal and increased T2-weighted image signal, consistent with the diagnosis of osteolytic bone metastases. **(E)** Post-operative radiograph of customized modular prosthesis implantation.

**Figure 4:**
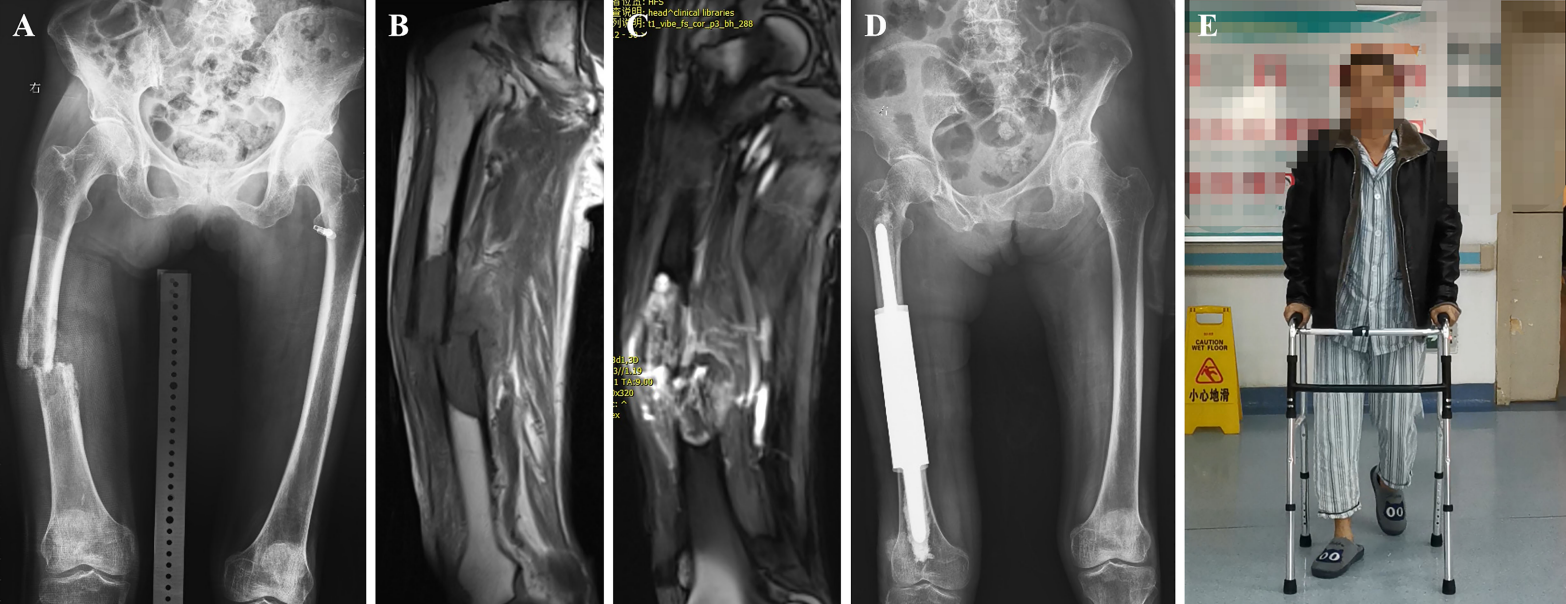
A case of metastatic lesion of the right femoral shaft with pathological fracture. **(A)** Radiograph showing osteolytic destruction of the right femoral shaft. **(B, C)** Long T1 and T2 signal shadows in the medullary cavity, local nodular changes, swelling of the surrounding muscle group, and increased signal. **(D)** Post-operative radiograph of customized modular prosthesis implantation. **(E)** Functional photo of the patient on the third postoperative day.

The medullary cavity was completely enlarged (**Figure 2C**), and the prosthesis was placed (**Figure 2D**). The bone marrow cavity was filled with bone cement and reset according to the designated normal limb force line. The prosthesis stalk in the fixed region of the medulla was at least 5 cm long. To position the prosthesis correctly, the medullary cavity was filled with a prosthesis stem coated with bone cement (**Figure 2E**). Once the bone cement cooled and dried, the connecting piece was secured with two screws, and the segmental prosthesis was then attached (**Figure 2F**). The extracted bone was submitted to a pathologist for analysis. A negative pressure drainage tube was inserted after full hemostasis, and the surgical incision was stitched together layer-by-layer. In **Figures 3**, **4**, two typical instances of femoral shaft metastases after en bloc excision and implantation of a specially designed modular prosthesis are shown.

### 2.6 Post-operative treatment

A negative pressure drainage tube was typically installed for 48 h and withdrawn when the daily discharge dropped below 50 mL. Analgesia, anticoagulant treatment, and postoperative infection control were frequently administered. A variety of post-operative systemic therapies, including radiotherapy, chemotherapy, hormone therapy, biotherapy, and immunotherapy, were used, depending on the systemic health of the patient and the features of the underlying metastatic tumor. Bisphosphonates or denosumab were administered for the management of bone pain and prevention of skeletal-related events.

### 2.7 Outcome assessment

The amount of intraoperative blood loss, surgery time, wound healing time, postoperative infection, internal fixation loosening or fracture, and re-fracture were recorded. After surgery, distant metastasis and local recurrence in the affected limb were routinely monitored.

Pre and post surgery (at 3, 6, and 12 months), the severity of pain was assessed using the visual analog scale (VAS), with a high score denoting severe discomfort (17). Lower limb function was assessed using the Musculoskeletal Tumor Society (MSTS) functional score, with a total score of 30; a high score indicates good function of the affected limb (18). A high score implies good limb function in the International Society of Limb Salvage (ISOLS) rating system (19). The Karnofsky Performance Status (KPS) scale was used to evaluate functional status; a high score indicates good functional health (20). The Nottingham Health Profile (NHP) scale was used to measure quality of life; a low score suggests minimal functional impairment and a good quality of life (21).

### 2.8 Statistical analysis

SPSS (version 21.0; IBM Corp., Armonk, NY, USA) was used for the statistical analysis. Using the paired sample t-test, the VAS pain, functional, and quality of life scores were compared. Statistical significance was set at *P*<0.05.

## 3 Results

**Table 1** lists the traits of the study participants. Each patient underwent an effective surgery and had stable vital signs throughout the procedure. Following surgery, post-operative pathology findings revealed bone metastases despite total removal of all tumors. The osteotomies ranged in length from 9 to 16 cm (mean, 11.72 cm), and the surgical duration ranged from 90 to 150 min (mean, 115.00 min). Patients were monitored for 12–62 months (mean, 25.28 months). No issues emerged during the observation period, except for two patients’ aseptic prostheses becoming looser.

**Table 1:** Clinical characteristics of patients.

Case no.	Sex	Age, years	Pathological fracture	Follow-up time, month	Primary site of metastases	Surgical duration, min	Osteotomy length, cm	First time of postoperative ambulation, days	Local recurrence	Survival time, month
1	Female	63	No	32	lung cancer	120	10	4	No	32
2	Male	54	No	28	breast cancer	100	12	3	No	21
3	Male	68	No	45	lung cancer	110	15	5	No	38
4	Female	63	Yes	37	kidney cancer	120	11	4	No	26
5	Male	72	No	12	thyroid cancer	90	10	6	No	9
6	Female	56	Yes	18	breast cancer	110	9	5	No	18
7	Male	69	No	15	lung cancer	140	10	4	No	15
8	Male	64	No	26	kidney cancer	100	12	4	No	10
9	Male	71	No	18	lung cancer	120	14	5	No	7
10	Female	62	Yes	16	kidney cancer	130	10	3	No	11
11	Male	79	No	22	stomach cancer	115	16	6	No	19
12	Female	72	No	17	thyroid cancer	150	11	5	No	17
13	Male	58	Yes	62	lung cancer	125	14	4	No	62
14	Female	61	No	25	cervical cancer	105	12	5	No	25
15	Male	66	No	18	lung cancer	100	13	7	No	18
16	Male	75	No	26	lung cancer	120	10	3	No	17
17	Female	71	Yes	15	colon cancer	110	12	5	No	8
18	Male	63	No	23	kidney cancer	105	10	4	No	23

**Figure 5:**
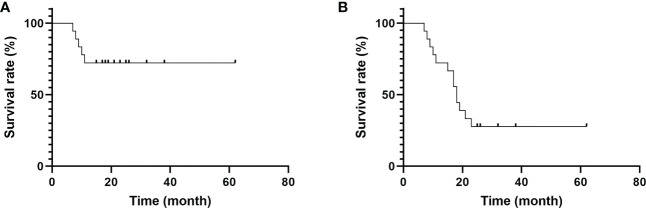
Survival time of patients. **(A)** The 1-year survival rate was 72.22%. **(B)** The 2-year survival rate was 27.78%.

The VAS and NHP scores decreased at 3, 6, and 12 months after surgery; however, the MSTS, ISOLS, and KPS scores increased, and the changes were statistically significant (*P*<0.05) (**Table 2**). The survival period was between 7 and 62 months (mean, 20.89 months), and the 1-year and 2-year survival rates were 72.22% and 27.78%, respectively (**Figure 5**).

**Table 2:** Comparison of preoperative and postoperative pain, functional status, and quality of life.

Item	Preoperative^*^	Postoperative third month	Postoperative sixth month^**^	Postoperative twelfth month^***^
Pain degree				
VAS score	8.54 ± 1.02	4.38 ± 0.57	2.38 ± 0.52	2.45 ± 0.22
Limb function				
MSTS score	22.17 ± 1.75	27.56 ± 1.98	28.28 ± 1.56	28.75 ± 2.13
ISOLS score	21.36 ± 1.06	25.69 ± 1.32	27.91 ± 1.31	28.19 ± 1.72
Life quality				
KPS score	61.83 ± 5.38	75.98 ± 5.40	77.58 ± 2.91	78.87 ± 1.72
NHP score	290.48 ± 28.56	226.42 ± 18.57	195.76 ± 23.18	195.26 ± 17.93

VAS, visual analogue scale; MSTS, musculoskeletal tumor society system; ISOLS, international society of limb salvage; KPS, karnofsky perfor mance status; NHP, nottingham health profile. ^*^: the postoperative third, sixth, and twelfth months compared to the preoperative, P<0.05; ^**^: compared to postoperative third month, P>0.05; ^***^: compared to postoperative sixth month, P>0.05.

## 4 Discussion

Patients with local tumor control, close pathological fractures, or failure of preventive internal fixation are candidates for whole-segment excision of primary or metastatic long-shaft malignancies (10, 22). Following a major resection of a diaphysis tumor, reconstructive techniques include the placement of massive allografts or autografts, replantation of inactive tumor bone, distraction osteogenesis, and insertion of segmental prostheses (23–27). Large allograft segments are immobilized during allograft implantation using intramedullary nails or steel plates (28). If the transplant is successful, no future revision surgery is required because the bone may permanently fuse with the allograft. Additionally, the transplanted allograft bone may cling to the repaired soft tissue, improving the post-operative stability (29). However, allografts have several major disadvantages, including allograft or residual bone fracture, graft rejection, non-union or poor matching between the allograft and autologous bone, and allograft non-union (30, 31). Additionally, even without a rejection reaction, the transplanted autologous bone may be unable to support weight for a considerable amount of time following the procedure, severely impairing the quality of life and reducing the function of the damaged limb (32). This approach cannot be used to reconstruct a significant backbone defect and carries the risk of graft breakage (33). After receiving inactivation treatment, the tumor tissue from the bone that constitutes the tumor segment is removed and replanted in its original location, restoring the continuity of the limb (34). However, it has drawbacks such as wound non-union, infection, fracture non-union, and replanted bone fracture, which have resulted in this kind of surgical method to be gradually abandoned (35). Distraction osteogenesis is a lengthy treatment that does not promote functional recovery or post-operative radiotherapy, carries the potential risk of needle tract infection, and is inappropriate for patients with metastatic disease (36).

The broad resection and repair of diaphysis tumors have recently used intercalary prosthesis implantation owing to the rapid development of biomaterials, biomechanics, iconography, internal fixation technology, and other procedures (10, 37, 38). Intercalary prosthesis implantation is clearly superior to intramedullary needle fixation, allogeneic bone transplantation, external fixation, and other techniques in terms of resisting extrusion, bending, and twisting (6). In a previous investigation, intercalary prosthesis implantation did not cause graft fracture or fracture healing after autologous and allogeneic bone transplantation (39). Functional exercise can be guaranteed in the early post-operative period, and normal function of the affected limb can be restored relatively sooner as the prosthesis has enough strength to bear stress similar to normal bone tissue, provided that the post-operative anatomical force line of the limb is normal (6). The osteotomy plane can precisely reach the area that needs to be excised because of the prosthesis’s ability to be customized, thereby lowering the local recurrence rate (40). In the case of diaphysis repair after large-segment osteotomy, the duration of the intercalary prosthesis implantation procedure is similarly reduced. These are well-known advantages of using an intercalary prosthesis over other types of restorations. These findings show that installing a tailored modular prosthesis has the added benefits of less trauma and less procedure time.

After intercalary prosthesis implantation, problems include prosthesis loosening, prosthesis wear, and prosthesis fracture, with prosthesis loosening being the most significant (41). Deviation of the limb force line is caused by loosening of the prosthesis, which can negatively impact the quality of life and necessitate reoperation. When the residual diaphysis or prosthesis cavity stalk becomes shorter following osteotomy, resulting in uneven tension on the prosthesis, prosthesis loosening may develop. Otherwise, it would be impossible to use bone cement to secure the prosthesis (42). In the case of a short prosthesis cavity stalk, some researchers have inserted an external cortical plate for better fixation to prevent prosthesis loosening; however, its long-term effects are yet to be determined (10). Despite the high prevalence of prosthesis loosening following surgery, few patients require reoperation for this complication (16, 43). Huang et al. described 16 cases of femoral metastatic tumors with pathological fractures treated with intercalary prosthesis implantation, one of which developed aseptic loosening 7 months following surgery (10). Sewell et al. reported 18 cases of tibial cancer treated with intercalary prosthesis implantation, four of which exhibited aseptic loosening. The authors considered that a stronger rotational force, larger medullary void in the metaphysis, and problematic distribution of bone cement contributed to easy loosening of the prosthesis (42). In our investigation, no complications occurred throughout the follow-up period, except for aseptic prosthesis loosening in two patients; however, revision surgery was not performed because the patients’ function was satisfactory.

In determining the success or failure of a surgery, post-operative function is an essential factor. Several biomechanical investigations (11) have proven that intercalary prostheses perform better than conventional fastening systems under various types of loading (four-point bending, torsion, and compression). Intercalary prosthetic repair is advantageous for patients with metastatic diaphyseal malignancies because of the advantages of instant stability, preservation of surrounding joints, and early return of function, according to research employing intercalary prostheses (9, 12, 16, 37, 40, 41). The MSTS score is used to evaluate the functional status of the musculoskeletal system of the skeleton after tumor removal and repair. Obtaining an adequate knowledge of surgical efficacy requires both subjective and objective post-operative evaluations. Ahlmann et al. retrospectively evaluated the clinical efficacy of intercalary prosthesis implantation in six patients with diaphyseal bone tumors, with a mean follow-up period of 21.6 months, and reported an average MTST score of 27 points, indicating that 90% of the functional status was restored (40). Abudu et al. reported the clinical outcomes in 13 cases of tibial and femoral diaphyseal tumors treated with intercalary prosthesis implantation; at the most recent follow-up, 84% of the patients’ function had been restored (44). The average post-operative MSTS score after intercalary prosthesis implantation for humeral malignancies, as reported by McGrath et al. (43), suggested 77% restoration of the patients’ functional status. In our study, the MSTS scores at 3, 6, and 12 months post-operatively were, 27.56 ± 1.98, 28.28 ± 1.56, and 28.75 ± 2.13, respectively. The three-dimensional printed prosthesis has a stronger bone integration effect and is worth looking forward to. The host bone is closely embedded with the prosthesis to achieve immediate stability, the microporous layer on the surface of the prosthesis is fused with the host bone, enabling long-term stability of the prosthesis (8, 9).

In the treatment of bone metastases, multimodal therapy is emphasized to prevent the progression of pain and skeletal-related events, and individualized treatment has become the direction of future development (45, 46). A multidisciplinary team of professionals in the diagnosis and treatment of bone tumors should select the most appropriate treatment strategy based on the patient’s unique condition, pathological type, metastasis, life expectancy, and family financial standing (47). In our study, the median survival time was 20.89 months, while the rate of complications was only 11.11%; the lower complication rate is more appropriate for patients with bone metastases who have a limited survival time.

## 5 Conclusion

For the treatment of femoral shaft metastases, en bloc resection and customized modular prosthesis implantation can reduce pain, improve limb function, and improve the quality of life. However, owing to the lack of a control group and the small sample size in our study, their efficacy should be tested further. Additionally, owing to the great variation in patients and primary tumors, it is difficult to generalize accurate and reliable universal principles and conclusions.

## Data availability statement

The original contributions presented in the study are included in the article/supplementary material. Further inquiries can be directed to the corresponding authors.

## Ethics statement

The studies involving human participants were reviewed and approved by the institutional review board of our hospital. The patients/participants provided their written informed consent to participate in this study. Written informed consent was obtained from the individual(s) for the publication of any potentially identifiable images or data included in this article.

## Author contributions

FP and ZZ performed the study and wrote the manuscript. FP and ZZ made substantial contributions to conception and design of the study. ZS and YY were responsible for the design of the study. YY and WW analyzed the study data. JF and FC assisted with the statistical analysis. YY and FC critically revised the manuscript, provided final approval of the version to be published and made substantial contributions to conception and design. All authors contributed to the article and approved the submitted version.
